# MRI-driven design of customised 3D printed gynaecological brachytherapy applicators with curved needle channels

**DOI:** 10.1186/s41205-019-0047-x

**Published:** 2019-05-16

**Authors:** Rianne C. Laan, Remi A. Nout, Jenny Dankelman, Nick J. van de Berg

**Affiliations:** 1 BioMechanical Engineering, Delft University of Technology, Delft, The Netherlands; 2 Radiation Oncology, Leiden University Medical Center, Leiden, The Netherlands; 3 Radiology and Nuclear Medicine, Erasmus MC, Rotterdam, The Netherlands

**Keywords:** Brachytherapy, Gynaecology, Personalised healthcare, Medical devices, Needle steering, Additive manufacturing

## Abstract

**Background:**

Brachytherapy involves placement of radioactive sources inside or near the tumour. For gynaecological cancer, recent developments, including 3D imaging and image-guided adaptive brachytherapy, have improved treatment quality and outcomes. However, for large or complex tumours, target coverage and local control with commercially available applicators remain suboptimal. Moreover, side effects are frequent and impact on quality of life. This signifies that brachytherapy treatment conformity can improve. Therefore, the aim of this study is to develop 3D printed personalised brachytherapy applicators with a custom vaginal topography and guided needle source channels, based on the patients’ anatomy.

**Methods:**

Customised applicators were derived from MRI data of two gynaecological cancer patients. Needle channels were planned by the Radiation Oncologist during image segmentation. Applicators contained multi-curved channels for 6F needles (ProGuide, Elekta) and were manufactured using a digital light processing-based 3D printer. Needle channel *radius constraints* were measured by analysing needle insertion forces in a 3D printed template, and imposed on the designs.

**Results:**

Two customised needle applicators are presented. Interstitial needle channels have tapered ends to increase needle protrusion angle accuracy. Additional structures were included to serve as anchor points in MR images for applicator and needle modelling and reconstruction during treatment planning. An insertion force analysis yielded a radius constraint of 35 mm to minimise the risk on needle jamming or buckling. For radii larger than 50 mm, no differences in insertion forces were found.

**Conclusion:**

A novel method to design and produce vaginal topography-based 3D prints for personalised brachytherapy applicators, derived from patient MRI data, is presented. The applicators include curved needle channels that can be used for intracavitary and guided interstitial needle placement. Further spatial optimisation of brachytherapy source channels to the patient anatomy is expected to increase brachytherapy conformity and outcome.

## Background

With an estimated 570.000 new cases and 311.000 cancer related deaths in 2018, cervical cancer is the fourth most common cause of cancer and cancer related deaths worldwide [1]. Brachytherapy (BT) is a key component in the curative treatment of cervical cancer [2]. In addition, gynaecological BT is used for the treatment of recurrent cancer in the vagina and as adjuvant therapy to reduce post-hysterectomy vaginal recurrences. Brachytherapy delivers radiotherapy locally, inside or near the tumour. This is achieved by guiding radioactive sources through channels in an applicator in the vaginal or uterine cavity (intracavitary applicators) or directly into tumour containing tissue (interstitial needles). Treatment planning of source dwell times and positions determines the dose distribution. An optimal BT treatment plan has high conformity, indicating an exact overlap of the target volume and prescribed isodose [3]. High conformity results in optimal target coverage and local tumour control, while minimising dose absorbed by surrounding healthy tissues, i.e. organs at risk (OAR). Personal and societal impact of treatment optimisation is crucial, as 5-year survival rates are at 65% and the majority of women are in their early decades of life [4].

Recently, substantial steps were made to improve radiation conformity, including the introduction of 3D imaging (CT/MRI) and the subsequent adaptation of BT treatment planning to the individual patients’ anatomy, i.e. Image Guided Adaptive Brachytherapy (IGABT). Yet, target coverage and local control remain suboptimal for larger tumours with extensive paravaginal or parametrial involvement (stage IIIA: 71%; IIIB: 75%) [4, 5]. Moreover, 12.5% of women reported substantial urinary toxicity, 25% experienced substantial bowel symptoms, and vaginal morbidity was frequently observed (53% mild, 19% moderate), impacting on (sexual) quality of life [6–9]. This underlines the importance of BT conformity to reduce toxicity and impact on quality of life.

**Fig. 1:**
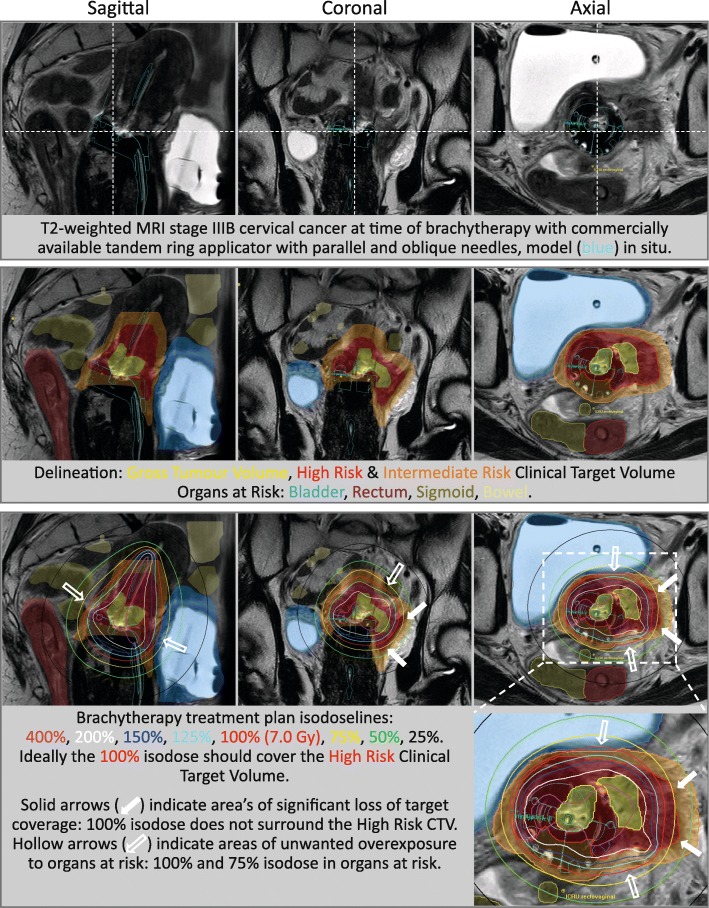
Limitations of an advanced standard applicator with parallel and oblique interstitial needles is demonstrated in a patient with a tumour involving the lateral parametrium. The delineation of relevant anatomy on T2 weighted MRI and the overlay of BT treatment plan isodose lines are shown. Underexposure of target volumes and overexposure of organs at risk are indicated by solid and hollow arrows

Currently, commercially available intracavitary applicators are one-size-fits-all products with fixed, rigid shapes and interstitial needle channels have fixed positions and angles. Most used intravaginal applicator shapes include ovoids, ring and cylinder. These shapes have been designed to obtain a historic standard pear formed dose distribution, while the thickness of these applicator parts kept high dose areas within the applicator. The main disadvantages are that these shapes do not align with individual anatomy, especially when this has been altered due to changes by cancer growth. Although the newest applicators have both parallel and oblique running needle channels, their positions and angels are fixed, hampering the ability to optimise the BT dose distribution remains insufficient (Fig. 1). Although target coverage is good in smaller tumours, considerable volumes of healthy tissue often receive an unnecessary dose. For larger tumours, especially those with substantial extensions in the distal parametria or lower (para)vagina, available standard applicators are particularly ill-adapted [10]. Supplementary free-hand or template based interstitial needles are required to improve target coverage. However, image guidance for accurate placement is often limited and conformity is subjected to the available techniques and skills of the Radiation Oncologist.

Recent developments in 3D printing have enabled a novel approach to BT in which applicators are patient-tailored by considering the individual target and healthy tissue volumes. During adjuvant, recurrent and primary BT, this approach can offer advantages for reliable applicator positioning within and between fractionated BT treatments [11], targeting lesions near or behind tissue folds [12], introducing curved needle channels and minimising the number of needles required [13], and enabling proficient treatment for patients with lesions in low-incidence locations, e.g. involving the lower (para)vagina or distal parametrium.

Several groups have developed personalised applicators, but have focussed either on intracavitary applicators, or on guided interstitial needle angles. The best known example of customised applicators is the vaginal mould technique, as described by Magné et al. Applicators were produced in a casting process with cervicovaginal impressions on the basis of alginate liquid pastes [11]. The intracavitary applicators were considered low-cost alternatives with a good patient tolerance. Huang et al. used 3D printed individual templates for needle guidance in head and neck BT, which resulted in an accurate transition from pre-planned to placed needle locations [14]. In various studies, 3D printing techniques have been used to improve the diametrical fit of intracavitary vaginal cylinder applicators [15–17]. Sethi et al. evaluated custom-fit cylinders for three patients that could not be treated adequately with commercial applicators [16]. The 3-D printing material used, PC-ISO, was biocompatible (ISO-10993 and USP Class VI) and gamma and EtO sterilisable. Interstitial needles were placed under transrectal ultrasound (TRUS) guidance. Lindegaard et al. developed 3D printed tandem-ring implants with customised needle channel locations [18]. Pre-planning, data processing and production were performed in-house within 3 days. Two studies have reported intracavitary vaginal topography-based prints using computed tomography (CT) data [13, 19]. The applicator developed by Wiebe et al. included curved intracavitary needle channels and consisted of two dove-tail connected parts to facilitate device insertion and removal [19].

To the authors knowledge, this is the first study to produce 3D printed vaginal topography-based applicators from MRI data. The applicators include multi-curved needle channels for both intracavitary and guided interstitial use. The article covers workflow-related aspects on data acquisition, segmented volume post-processing and instrument design, including an analysis of needle channel radius constraints.

## Materials and methods

**Fig. 2:**
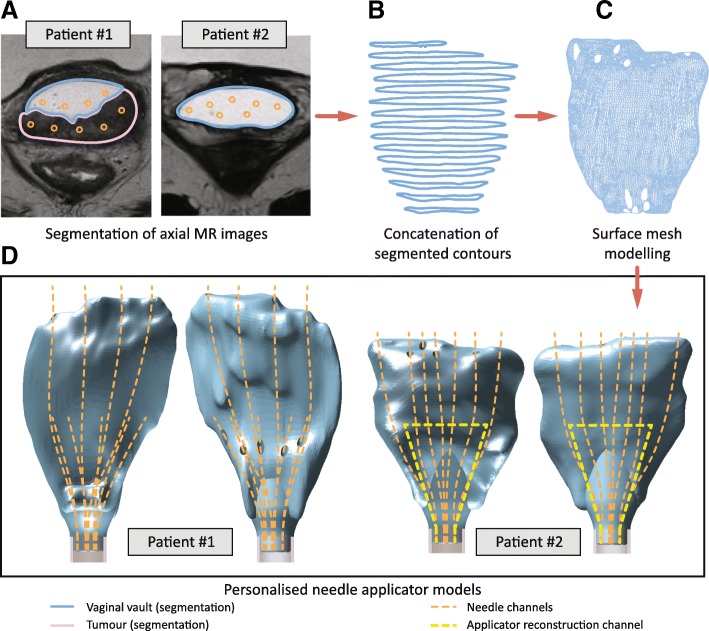
Illustration of data processing steps, including **a**) segmentation of MR images, **b**) concatenation of segmented contours, **c**) surface mesh modelling, and **d**) applicator designs for two patients. The designs consist of a uniform cylindrical base and a vaginal topography-based needle template. The template has curved needle channels for intracavitary and interstitial use

Development of customised applicator designs was based on pre-BT MRI data of two recurrent gynaecological cancer patients. Prior to the pre-BT MRI, aqueous gel was manually injected into the vagina to augment the distention and visibility of the vaginal vault [20]. After image acquisition, treatment planning software (Oncentra, Elekta, Sweden) was used to segment the vaginal vault, target volume and OARs (Fig. 2a). Desired channels for 6F needles with obturator (ProGuide, Elekta, Sweden) were indicated manually at this stage. Contours were saved in DICOM RT-structure files.

Applicators consist of a personalised needle template, merged with a uniform instrument base. The personalised template was formed by concatenation of segmented contours (Fig. 2b) of the vaginal vault and needle channels. A 3D geometrical reconstruction of the vaginal vault was created by surface mesh modelling (Fig. 2c). This was done in 3D Slicer [21] by importing the DICOM RT tructure file (SlicerRT extension) and processing contours by respectively selecting a binary labelmap and a closed surface representation (smoothing factor: 0.5). The 3D surface model was exported as STL file. Planned needle channels were extracted from the RT-structure file and stored in a TEXT file using MeVisLab (2.7.1, MeVis Medical Solutions AG, Germany), using the modules CSOManager, CSOConvertToXMarkerList and XMarkerListToFile. Coordinates were post-processed in MATLAB (R2017b, MathWorks, USA) to create smooth interpolated splines (interp1). A computer-aided design program (SolidWorks, Dassault Systemes, USA) was used to make a uniform instrument base. The vaginal topography STL was imported as a solid and joined to this uniform base (Fig. 2d). The needle channel coordinates (TEXT file) were opened as a PointCloud in the same file. Needle splines were reconstructed in 3D sketch mode and swept cuts were created with a cross-sectional diameter of 2.6 mm. The interstitial needle channels were tapered to a 2.2 mm diameter to increase the directional accuracy of protruding needles.

All parts were 3D printed from a liquid photopolymer resin (R5, EnvisionTEC, Germany), using a digital light processing (DLP)-based printer (Perfactory 4 mini XL, Envisiontec, Germany), and a layer height of 50 μm. The slim uniform base (diameter 12 mm) was chosen to minimise stress levels at the vaginal introitus. For patient No. 2, two channels were interlinked within the applicator (Fig. 2c, yellow dotted line), forming a loop that can be filled with water or aqueous gel during MRI. This loop provides anchor points for applicator and needle modelling and reconstruction and BT treatment dose planning.

**Fig. 3:**
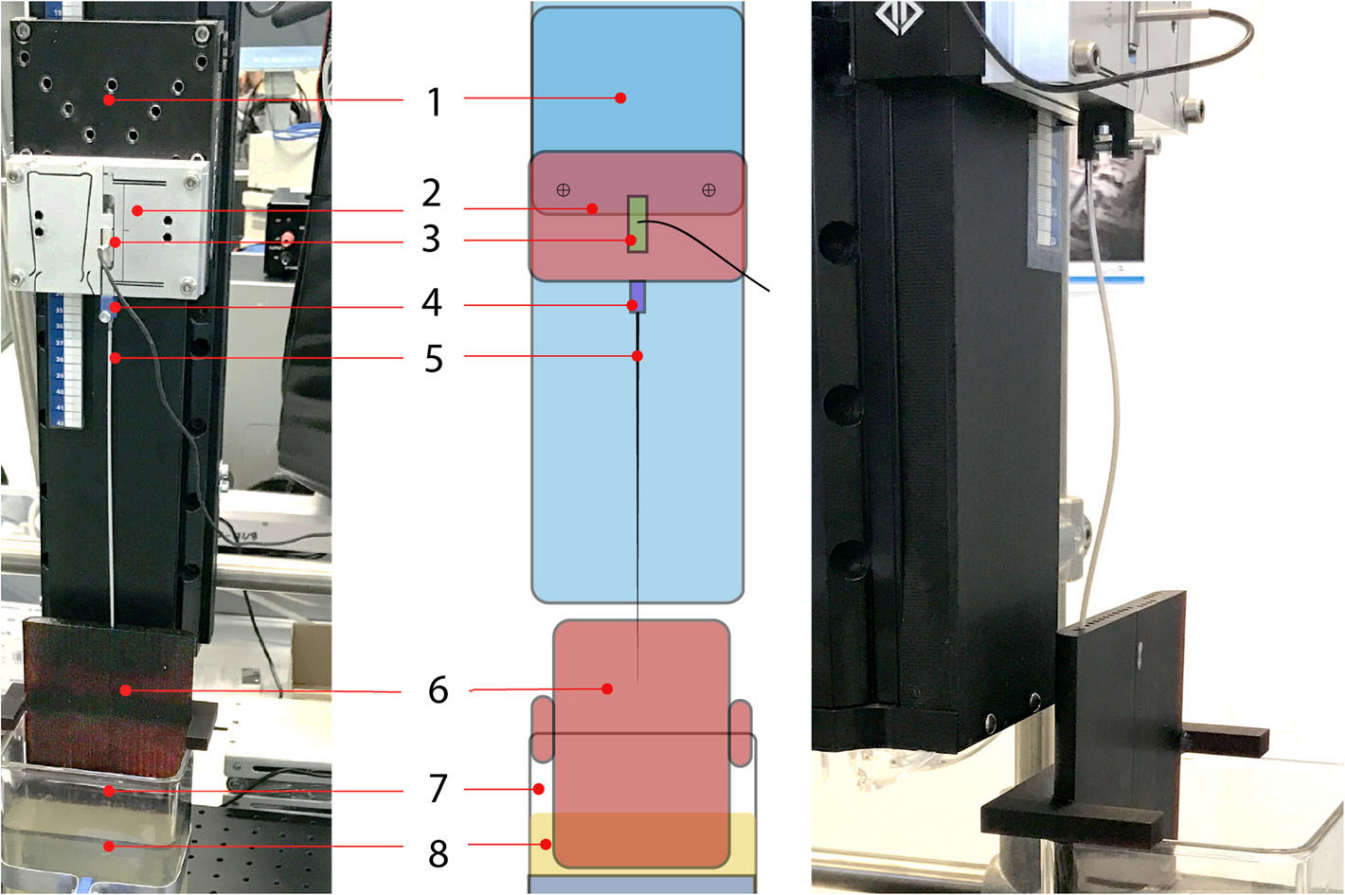
Experimental platform used to compare needle insertion forces in curved channels. Illustrated are the 1) linear stage, 2) force sensor mounting block, 3) force sensor, 4) needle fixture, 5) needle, 6) needle template with curved channels, 7) phantom container, and 8) phantom. The photograph on the right shows a buckled needle

Required needle insertion forces were expected to inversely relate to the radius of curvature. Design constraints for curved channels were assessed experimentally with a 3D printed needle template (Fig. 3). The template contained an array of 2.6 mm diameter needle channels, with channel radii (*r*) ranging between 20 and 75 mm, with intervals of 5 mm. The template was printed in its vertical position. The channels bridged a wall thickness of 5 mm. During the experiment, the template was embedded in phantom material (10 wt% gelatin in water, Dr. Oetker, Germany). Brachytherapy needles with blunt and sharp tips were inserted at 5 mm/s, using a linear stage (PRO-115, Aerotech, USA). The axial insertion forces were measured with a load cell (LLB130, Futek, USA).

In the experimental design, needle channel radii were randomised per tissue phantom. In sequence, ten gelatin phantoms were used to assess insertion forces for two tip types (blunt and sharp) and five repetitions. Force data were collected successfully for channels with 35 ≤ *r* ≤ 75 mm. For *r* < 35, needles buckled and the insertions were aborted (Fig. 3).

Force data were processed with a zero-phase moving average filter in MATLAB (kernel size of twenty). The average forces were computed for each channel radius level. In addition, force maxima, medians, 25th and 75th percentiles were stored as summary statistics. To evaluate differences among conditions, a two-way ANOVA was performed, followed by a Tukey-Kramer multiple comparison evaluation (significance level α = 0.05).

## Results

**Fig. 4:**
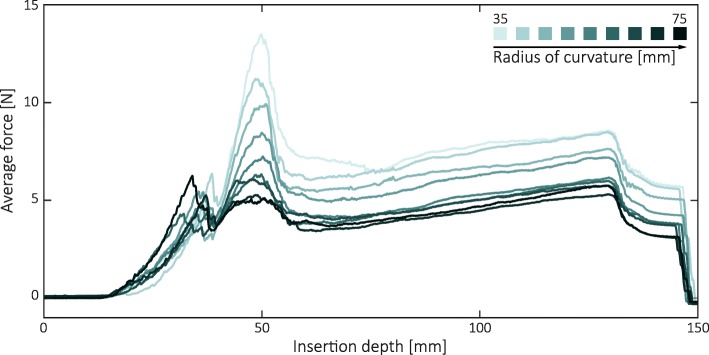
Illustration of average insertion forces for the blunt needle type, sorted by radius of curvature. A similar response was seen for the sharp needle type. The force peaks occurred within the applicator

Averaged forces versus needle insertion depth were sorted by needle channel radius (Fig. 4). Force maxima occurred within the applicator, at an approximate insertion depth of 50 mm. For *r* < 35 mm, buckling occurred when axial forces exceeded approximately 14 N. Since forces of a similar magnitude were observed for *r* = 35 mm, this radius was close to the testing limit of our platform. After the peak, forces dropped and gradually increased again as a function of inserted needle length propagating through phantom tissue. Force slopes run approximately parallel, but have offsets of different magnitudes. Overall, lower insertion forces could be attributed to higher channel radii.

**Fig. 5:**
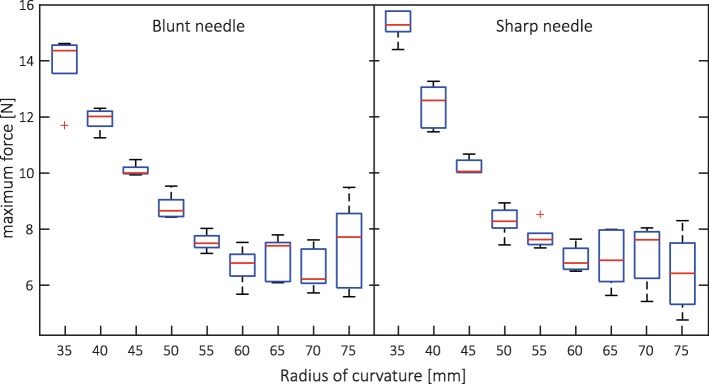
Maximum axial forces for BT needles during their insertion in curved applicator channels. Commonly used needle types with blunt and sharp tips were evaluated

Peak values of these force profiles are compared (Fig. 5), for both blunt and sharp needles. The boxplots indicate median values and 25th and 75th percentiles. The ANOVA results present a difference among radii (*p* < 0.001), no difference among needle types (*p* = 0.35) and no interaction effect (*p* = 0.14). The maximum forces differed from one another for all *r* ≤ 50 mm. No differences were found for *r* > 50 mm.

**Fig. 6:**
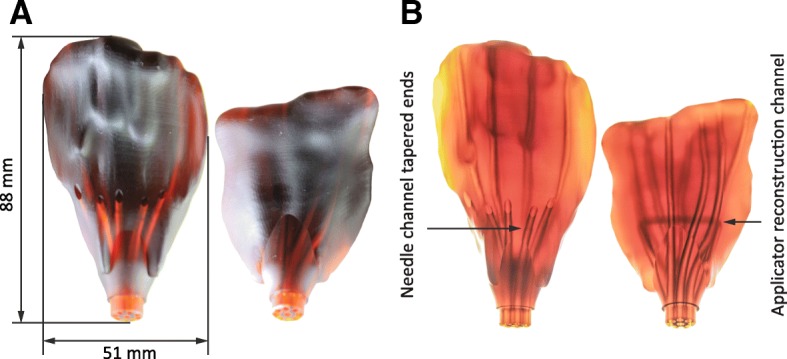
Photos of 3D printed gynaecological BT needle applicators (**a**) for the two included patients. Photos of the applicators against background (LED panel) lighting (**b**) reveal the internal needle channels, the tapered ends and the applicator reconstruction channel

Two 3D printed applicators, with needle radius constraints (*r*_*min*_ = 35 mm) were developed (Fig. 6a). Figure 6b shows the applicators illuminated by a LED panel. All interstitial needle channels contained tapered ends. One applicator included a reconstruction channel to assist applicator modelling and BT treatment dose planning in MR images.

## Discussion and conclusion

Methods to design and construct customised BT needle applicators from individual patient MRI data, as well as design examples for two gynaecological cancer patients, are presented. The applicators include curved needle channels for intracavitary and interstitial use. Enforcing needle deflections within applicators results in increased normal forces and friction. For high curvatures or multi-curved configurations this friction can become a dominant factor of the insertion force. At some point, needles may jam or buckle. In practice, the risk of buckling may be prevented by mechanical supports or by ‘pre-loading’ the needles in the applicator. However, when stick-slip friction occurs, force build-up may hamper the ability to precisely control needle displacements and assess positions [22]. Needle channel radius constraints are therefore crucial to meet procedural quality and safety demands. Needle channel radius constraints were assessed by experiment, studying insertion forces in a 3D printed needle template. A practical minimum radius of curvature in this assessment was 35 mm. However, if force feedback is considered an important factor in needle placement, channels with radii larger than 50 mm should be considered.

As needles were bundled at the slim uniform applicator base, curvature constraints directly interfered with the reachable space. In this work, needle channels were planned by the Radiation Oncologists. However, automated planning of needle source channels will be a next step in the development of customised BT applicators. This field can rely on a vast amount of literature on needle steering techniques [23]. For BT applicators, Garg et al. proposed a path planner that was based on rapidly-exploring random trees [13], although the selected minimum radius of curvature of 10 mm was not substantiated. Although this constraint differed from our findings, it should be noted that differences are expected when needles with different stiffness are used, e.g. other than 6F, or when applicators are printed with a dissimilar surface quality.

The strong relation between radius of curvature and maximum force (Fig. 5) indicates the dominant role of friction (needle-applicator interaction) in the insertion force. Needle-tissue interaction, including tissue cutting forces, will also contribute to total insertion force. Although an effect of tip type (sharp/blunt) was expected during interstitial needle use [24], this was not visible in the data. Presumably, higher quality tissue phantoms or ex-vivo experiments are needed to study these effects in more detail.

Quality management, risk analysis and patient comfort are crucial and interrelated factors that need to be addressed to proceed from preclinical to clinical work. For example, dosimetric impact of various 3D printed layers and materials should be better understood. Ricotti et al [17]. found no effect of infill percentage of ABS on dose distributions in 3D printed vaginal cylinders. However, similar dose measurements should be performed for liquid photopolymer resins and other 3D printing materials in order to compare material adequacy and their potential impact on dose distributions for BT applications. Printing techniques also affect geometrical tolerances, reproducibility and surface quality. Production factors that should be controlled include circularity of channels to ensure needle access and material roughness to reduce friction and improve patient comfort. Quality and safety standards also concern patient comfort during applicator insertion and removal. This can be resolved by applicator design. For instance, Wiebe et al [19]. proposes to divide custom applicators in two dove-tailing parts.

The extent to which BT dose distributions can be optimised by treatment planning relies on the strategic spatial distribution and accurate placement of intracavitary and interstitial source channel needles. The customisation of applicators to the patient’s anatomy is an emerging field that aims to qualitatively advance these needle placement tasks. Additive manufacturing is currently an ideal facilitator for customisations and may even decrease product costs [25]. At present, device customisation took approximately 4 h, but this will likely change with imminent developments in automating image segmentation, path planning and data type conversions. Future adaptive systems may include actively adjustable applicators, e.g. that rely on TRUS-based tuning of needle channels. This may be realized by semi-flexible 3D prints and embedded compliant and echogenic mechanisms [26], controlled in either a manual or soft-robotics setting. The same type of flexible structures may be used to simplify applicator introduction or removal.

In conclusion, a novel approach to design and produce personalised vaginal topography-based 3D prints for BT needle applicators, derived from patient MRI data, has been developed. Customised applicators are expected to stabilise applicator positions, improve lesion access, optimise spatial needle channel distributions and enhance access to less frequent tumour locations, thereby improving BT treatment conformity, increasing local control in large extensive tumours and decreasing side effects and their impact on quality of life.

## Abbreviations

BT: Brachytherapy
CT: Computed tomography
DLP: Digital light processing
HDR: High dose rate
IGABT: Image guided adaptive brachytherapy
MRI: Magnetic resonance imaging
OAR: Organs at risk
